# Clinical value of multi-gene testing in distinguishing benign and malignant thyroid nodules

**DOI:** 10.1097/MD.0000000000035960

**Published:** 2024-01-26

**Authors:** Murui Zhang, Xiaotong Hu, Lunming Liu, Yihong Wang, Junchang Jiang, Hui Li, Weiqiang Fei, Tingting Zhong, Zhinong Jiang

**Affiliations:** a Department of Pathology, Sir Run Run Shaw Hospital of Zhejiang University College of Medicine & Sir Run Run Shaw Institute of Clinical Medicine of Zhejiang University, Hangzhou, China; b Zhejiang Chinese Medical University, Hangzhou, China.

**Keywords:** bethesda system for reporting thyroid cytopathology (BSRTC), fine needle aspiration (FNA), gene mutation, NGS, thyroid nodules

## Abstract

**Background:**

The newly released 2022 WHO Classification of Neuroendocrine Neoplasms (version 5) and a recent update on thyroid tumor classifications have emphasized genetic testing to an unprecedented level. Fine needle aspiration (FNA) has been widely applied for the preoperative diagnosis of thyroid nodules. However, it is limited mainly to testing for a single gene-BRAF^V600E^, whereas multi-gene testing data are scarce, especially in the Asian population. This study aimed to explore the clinical value of multi-gene testing in the differential diagnosis of benign and malignant thyroid nodules based on the 2023 Bethesda System for Reporting Thyroid Cytopathology (BSRTC).

**Methods:**

A total of 615 thyroid nodules underwent ultrasound-guided fine-needle aspiration cytology (FNAC) were collected from Sir Run Run Shaw Hospital, Zhejiang University School of Medicine. The next-generation sequencing platform was applied for multi-gene testing. A panel of well-recognized commonly mutated genes in thyroid cancer were analyzed, including BRAF^V600E^, KRAS, NRAS, HRAS, TERT, TP53, PAX8/PPARG, CCDC6/ RET and NCOA4/ RET.

**Results:**

Gene mutations were identified in 324 nodules (52.7%), with BRAFV600E being the most prevalent driver gene alteration observed in this cohort (233/324; 79.1%), followed by RAS (77/324, 23.8%). The overall malignancy rate of gene mutations was 89.7% in our cohort, of which the lymph node metastasis rate was 45.3%. The combination of multi-gene testing and cytology resulted in 89.3% sensitivity, 95.2% specificity, 98.9% positive predictive value, 64.5% negative predictive value and 90.3% accuracy, which were significantly higher than those from mere cytology (sensitivity 68.6%, specificity 87.5%, positive predictive value 95.9%, negative predictive value 39.8%, accuracy 72.2%).

**Conclusions:**

Multi-gene testing could substantially enhance the detection rate of malignant thyroid nodules and protect patients with benign nodules from unnecessary surgeries. Multi-gene testing provides a valuable reference for individualized preoperative decision-making, which may serve as a crucial method for postoperative treatment and prognosis assessment.

## 1. Introduction

Thyroid neoplasms are common in the endocrine system, representing the most commonly diagnosed head and neck tumors. In the past few decades, the total incidence rate of thyroid neoplasm has increased about 2-fold and comprised 2% of the cancers.^[1]^ Thyroid nodules are clinically prevalent and can be detected in approximately 20% to 76% of normal people by ultrasound techniques.^[2]^ As the development of detecting and surgical techniques, an increasing number of thyroid nodules has been found as malignancy. In this context, early screening and differentiation of benign and malignant thyroid nodules are decisive for clinical decision-making. Currently, ultrasound-guided fine-needle aspiration cytology (FNAC) is widely applied in the preoperative diagnosis of benign and malignant thyroid nodules. It is interpreted based on The Bethesda System for Reporting Thyroid Cytopathology (BSRTC). However, a definitive diagnosis still cannot be achieved via the FNA for 15–30% of nodules.^[3,4]^

With the recent advancement of molecular techniques to diagnose thyroid neoplasm, amounting evidence has proved that BRAF^V600E^ -based FNA distinctly increases the detection rate of BRAF-mutated associated thyroid neoplasm.^[5,6]^ However, studies on multi-gene testing have been mainly limited to the western population^[7–10]^ and are less common in Asians. It is worth noting that different races may vary in molecular alterations in this type of tumor. Previous research showed that the common mutated gene: BRAF^V600E^, ranged from 29% to 83% (mean: 45%) in papillary thyroid cancer (PTC),^[11,12]^ with higher alternation frequency in Asian people (70–80%)^[13,14]^ and lower mutations in the Americans (40–50%),^[15]^ although the rate has increased in the recent decade.^[16]^

Given the growing need for individualized diagnosis and therapy, neither mere cytology nor single-gene testing is satisfactory for clinical practice. Herein, we established a 9-gene mutation panel (BRAF^V600E^, KRAS, NRAS, HRAS, TERT, TP53, PAX8/PPARG, CCDC6/ RET and NCOA4/ RET) to sketch the gene mutation profile of thyroid neoplasms and to explore the diagnostic value of multi-gene testing in Chinese population based on BSRTC.

## 2. Materials and methods

### 2.1. Patients

**Figure 1.**
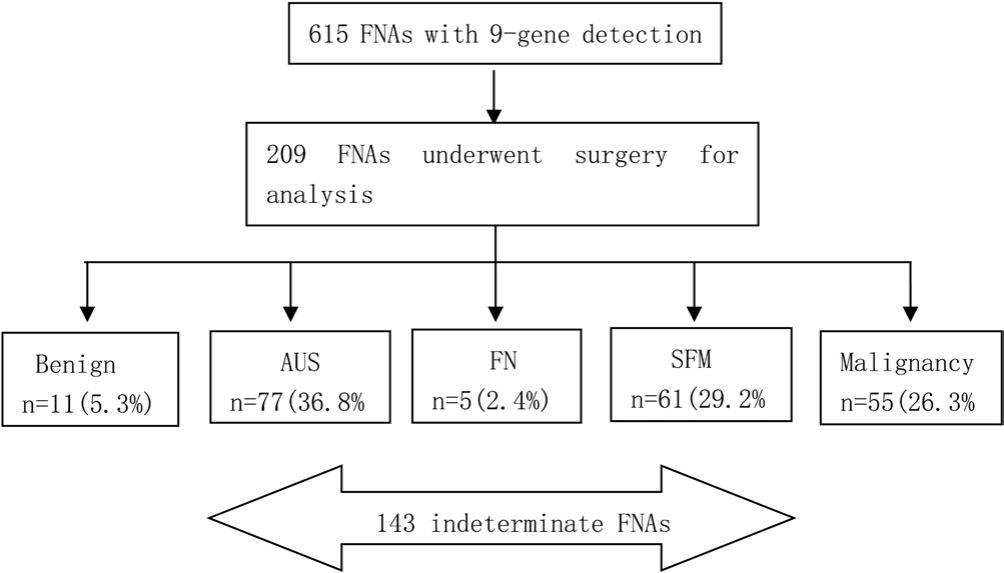
Schematic representation of the study design. Study subjects and number of ultrasound-guided FNA specimens collected during the course of the study. All of the 209 eligible FNAs with a valid 9-gene detection result, a matching histopathologic and cytologic reference standard diagnosis were included in the analysis.

A total of 615 patients with thyroid nodules underwent ultrasound examination, ultrasound-guided FNA biopsy (FNAB), cytological examination and 9-gene mutation panel testing in Head and Neck Surgery of our hospital between April 2019 and February 2022 were included. All nodules were documented with complete clinical data, and 209 of them with histopathological diagnosis (excluding 1 parathyroid sample) were processed for further analysis (Fig. 1). All participants were fully informed of the FNA and gene testing procedures and signed the Informed Consent. This study was approved by Ethics Institutional Review Board of Sir Run Run Shaw hospital and was conducted according to ethical and institutional rules regarding research on patients and tissue specimens.

### 2.2. FNAB and cytology

FNAB was fulfilled by a sonographer and his assistant. Briefly, each nodule was punctured 3–5 times free of negative pressure in multiple directions using a 25 G needle, with a total of 3–4 needles. Smear samples corresponding to each needle were obtained, instantly fixed in 95% alcohol for 20 minutes and then stained and microscopically reviewed for pathology. All smear samples were diagnosed by 1–2 trained cytopathologists based on the 2023 Bethesda System for Reporting Thyroid Cytopathology, and were graded as Bethesda I-VI: nondiagnostic, benign, atypia of undetermined significance, follicular neoplasm (FN), suspicious for malignancy, and malignancy. The physicians selected the smears or circled the suspicious areas on the smears. The molecular technician then scraped the cells into a lysate-contained EP tube for further gene testing.

### 2.3. Nucleic acid extraction and gene testing

Following the standard procedure of the next-generation sequencing kit, gDNA and RNA were extracted from FNA samples. Single-nucleotide variants (SNV) sites of the 6 genes (BRAF^V600E^, KRAS, NRAS, HRAS, TERT, TP53) and fusion regions of the 3 fusion genes (PAX8/PPARG, CCDC6/ RET, NCOA4/ RET) were separately amplified and prepared as a library available for Illumina sequencing.

1. Nucleic acid was extracted using the Qiagen AllPrep DNA/RNA FFPE extraction kit following the manufacturer instructions (omitting the sample processing deparaffinization procedures). The concentration and quality of DNA/RNA were determined using the Nanodrop ND-2000 with the standards as below: DNA: OD260/OD280 = 1.8-2.1; RNA: OD260/OD280 = 1.9-2.0.
2. Gene testing was performed conforming to the standard procedure of next-generation sequencing. Qubit3.0 (Invitrogen) was used for preliminary quantification after library establishment, followed by cDNA synthesis, cDNA quality control, first round of cDNA and gDNA amplification, first round of cDNA and gDNA purification, second round of cDNA and gDNA amplification, second round of cDNA and gDNA purification (cDNA and gDNA libraries), determination of library concentration, monitoring of fragment length distribution, uploading and sequencing.
3. Bioinformatics procedures were as below: raw data filtering, quality assessment, alignment to a reference, evaluation of alignment results (SNV, Fusion).

Sequencing was performed using the Miseq Reagent Kit v2 (300 cycles)/MiniSeq High Output Reagent Cartridge (300 Cycles).

### 2.4. Statistical analysis

The sensitivity, specificity, positive predictive value (PPV); negative predictive value (NPV), and accuracy for the 9-gene mutation panel were calculated using SPSS (version 22.0; IBM Corporation, Armonk, NY, USA) and software (Version 4.1.1, http://www.r-project.org) for Windows.

## 3. Results

### 3.1. Mutation panel establishment

According to the US National Comprehensive Cancer Network guidelines for diagnostically significant genes and well-validated thyroid-related genes in published literature, 6 genes with point mutations, including BRAF^V600E^, NRAS (codons 12, 13, 59, 61), HRAS (codons 12, 13, 61), KRAS (codons 12, 13, 59, 61, 117, 146), TERT, TP53 (Arg273), and 3 gene with rearrangements, including PAX8/PPARG, CCDC6/ RET and NCOA4/ RET were obtained. Totally, a 9-gene mutation panel was established in this study.

Multi-gene testing was performed to examine DNA mutation and RNA fusion using multiplex PCR library on the Illumina high-throughput sequencing platform. The sequencing depth on average with respect to DNA SNV, small insertions/deletions was more than 1000×. The limit of detection for mutation frequency was 2%. The average sequencing depth in terms of RNA sequencing for fusions in internal reference genes was over 2000×. The reference genome sequence was human UCSC hg19 Feb.2009.

### 3.2. Results of multi-gene testing

In a total 615 FNA samples, 324 samples (52.7%) had the most prevalent gene mutations in BRAF (233/324, 71.9%), followed by RAS (77/324, 23.8%) majorly represented by NRAS (44/77, 57.1%) and KRAS (24/77, 31.2%). TERT mutation was detected in 2 samples, RET rearrangement as CCDC6/RET was found in 1 sample. Combined mutations were detected in 11 samples, including BRAF + RAS (n = 8), BRAF + TERT (n = 1), RAS + TERT (n = 1) and RAS + RET (n = 1). No mutations were found in TP53 and PAX8/PPARG arrangement (Table 1). Among the cases with BRAF mutations, 99.2% were defined as malignant PTC with 68.3% developed lymph node metastasis. RAS mutations was noted in 45.8% of malignancies, including 9 PTCs, 1 follicular thyroid carcinoma (FTC) and 1 noninvasive follicular thyroid neoplasm with papillary like nuclear features (NIFTP). There were 6 samples developed lymph node metastasis (6/11, 54.5%), including 5 NARS-mutated samples (5/6, 83.3%). The combination of BRAF and RAS mutations led to 100% malignancy (3 PTCs and 1 FTC), half of which had lymph node metastasis. The overall malignancy rate was 89.7%, of which 64.7% had lymph node metastasis.

**Table 1:** Proportion of histological groups with gene mutations.

Gene mutation	N(%)	Surgery(%)	Benign(%)	Malignant(%)	LNM(%)
BRAF	233 (71.9)	121 (51.9)	1 (0.8)- FA(1)	120 (99.2)- PTC(120)	82 (68.3)
RAS	77 (23.8)	24 (31.2)	13 (54.2)-FND(6),FA(6),OA(1)	11 (45.8)-PTC(9), FTC(1),NIFTP(1)	6 (54.5)
NRAS	44	15		4	0
KRAS	24	6		5	5
HRAS	7	2		1	0
NRAS + KRAS	1	1		1	1
NRAS + HRAS	1	0		0	0
RET	1 (0.3)	1 (100.0)	0	1 (100.0)- PTC(1)	0
TERT	2 (0.6)	2 (100.0)	1 (50.0)-FA(1)	1 (50.0)- FTC(1)	0
BRAF + RAS	8 (2.5)	4 (50.0)	0	4 (100.0)- PTC(3),FTC(1)	2 (50.0)
BRAF + NRAS	4	3		3	1
BRAF + KRAS	2	1		1	1
BRAF + HRAS	1	0		0	0
BRAF + KRAS + NRAS	1	0		0	0
BRAF + TERT	1 (0.3)	1 (100.0)		1 (100.0)- PTC(1)	0
RAS + TERT	1 (0.3)	1 (100.0)		1 (100.0)- PTC(1)	0
RAS + RET	1 (0.3)	1 (100.0)	1 (100.0)-FA(1)	0	0
Total	324	155 (47.8)	16 (10.3)	139 (89.7)	90 (64.7)

The molecular distribution of 615 FNAs and the results of postoperative pathological analysis.

FA = Follicular adenoma, FND = Thyroid follicular nodular disease, FTC = follicular thyroid carcinoma, LNM = lymph nodes metastasis, NIFTP = noninvasive follicular thyroid neoplasm with papillary-like nuclear features, OA = oncocytic adenoma, PTC = papillary thyroid carcinoma.

### 3.3. Gene mutations in histopathology

There were 209 nodule samples with histopathological diagnoses, and 155 (155/209, 74.2%) had gene mutations (Table 2). There were 37 (37/209, 17.7%) benign nodules, 3 (3/209, 1.4%) low-grade malignancies and 169 (169/209, 81.6%) malignancies. Correspondingly, gene mutations were detected in 16 (16/37, 43.2%) benign nodules, 1 (1/3, 33.3%) low-grade malignancy and 138 (138/169, 81.7%) malignancies. Among the benign nodules, follicular adenomas (FA) and thyroid follicular nodular disease (FND) were the most commonly entities which harbored RAS gene mutation (14/16, 87.5%). In the 3 low-grade malignancies, low-risk follicular cell-derived neoplasms including NIFTP accounted for the major part (2/3, 66.7%) with 1 harbored RAS mutation and the other as hyalinizing trabecular tumor (HTT) had not gene mutation. In the 169 malignancies, the vast majority were PTCs (164/169, 97%), 135 (135/169, 82.3%) out of which had gene mutations represented by BRAF mutation (125/135, 92.6%). In addition, there were 3 follicular tumors with mutations (including RAS gene mutation in 2 malignancies) and 2 oncocytomas (OCC) without any mutation.

**Table 2:** Gene mutation rates in different histopathological groups.

	Histological diagnosis	N (%)	Gene mutation+(%)
Benign tumors		37 (17.7)	16 (43.2)
	Thyroid follicular nodular disease(FND)	15	6 (RAS)
	Follicular adenoma(FA)	19	9 (1BRAF, 6RAS,1TERT,1RAS and RET)
	Oncocytic adenoma(OA)	3	1 (RAS)
Low risk neoplasms		3 (1.4)	1 (33.3)
	NIFTP	2	1 (RAS)
	Hyalinizing trabecular tumor(HTT)	1	0
Malignant neoplasms		169 (81.6)	138 (81.7)
	Papillary thyroid carcinoma(PTC)	164	135 (120BRAF,9RAS,1RET,3BRAFandRAS, 1BRAF and TERT,1 RAS and TERT)
	Follicular thyroid carcinoma(FTC)	3	3 (1TERT,1RAS,1BRAF and RAS)
	Oncocytic carcinoma(OCC)	2	0
Total		209	155 (74.2)

The molecular distribution of 209 FNAs with the results of postoperative histopathology.

NIFTP = Low-risk follicular cell–derived neoplasms include noninvasive follicular thyroid neoplasm with papillary-like nuclear features.

### 3.4. Gene mutation in cytology based on BSRTC

Based on Bethesda 2023 classification, 11 nodules (11/209, 5.3%) were diagnosed as benign (Bethesda class II), 143 (143/209, 68.4%) were undefined (Bethesda classes III/IV/V), and 55 (55/209, 26.3%) were diagnosed as malignant (Bethesda class VI) (Table 3). The benign nodules were pathologically proved as benign with a gene mutation rate of 36.4%. The malignant nodules were pathologically confirmed as PTC, and the gene mutation rate was 85.5% and mainly BRAF mutation (125/135, 92.6%). The results suggested that gene testing is less helpful in diagnosing of Bethesda class II/VI tumors in cytology. The mutation rate of samples with cytological uncertainty (Bethesda classes III, IV, and V) was 70.6%, and the malignancy rate was 81.8%. The sensitivity, specificity, positive predictive value, negative predictive value, and accuracy were 76.1%, 53.8%, 88.1%, 33.3% and 72.0%, respectively.

**Table 3:** Correlation between cytology, molecular, and pathology diagnosis in 209 FNA specimens.

Bethesda category	Gene mutation	Gene+(%)	Histological diagnosis	Malignant (%)
II	BRAF(1)	36.4	FA(1)	0
	RAS(3)		FND (3)	
	No mutation(7)		FND (5),FA(1),OA(1),	
III,IV,V	BRAF(73)	70.6	PTC(73)	81.8
	RAS(21)		PTC(9),FTC(1),NIFTP(1)FA(6),OA(1), FND (3)	
	RET(1)		PTC(1)	
	TERT(2)		FTC(1),FA(1)	
	BRAF and RAS(3)		PTC(2),FTC(1)	
	RAS and RET(1)		FA(1)	
	No mutation(42)		PTC(24),OCC(2),NIFTP(1),HTT(1),FA(9),OA(1), FND (4)	
VI	BRAF(47)	85.5	PTC(47)	100.0
	BRAF and RAS(1)		PTC(1)	
	BRAF and TERT (1)		PTC(1)	
	RAS and TERT (1)		PTC(1)	
	No mutation(5)		PTC(5)	

FA = follicular adenomas, FND = follicular nodular disease, FTC = follicular thyroid carcinoma, PTC = papillary thyroid cancer.

### 3.5. Comparison of multi-gene testing with cytology in diagnosis of benign and malignant nodules

The combination of multi-gene testing and cytology resulted in increased diagnostic sensitivity (68.6% to 89.3%), specificity (87.5% to 95.2%), positive predictive value (PPV) (95.9% to 98.9%), negative predictive value (NPV) (39.8% to 64.5%), resulting in a high accuracy as 90.3% (Table 4). The results indicate that further multi-gene testing can significantly improve the accuracy rate in diagnosis of benign and malignant thyroid nodules.

**Table 4:** Comparison of diagnostic performance between cytology and molecular testing (n = 209).

Diagnostic performance(%)	Cytology alone	Molecular test	Cyto + Molecular test
Sensitivity	68.6	81.3	89.3
Specificity	87.5	57.9	95.2
PPV	95.9	89.7	98.9
NPV	39.8	40.7	64.5
Accurary	72.2	77.0	90.3

The sensitivity, specificity, PPV, NPV, and accuracy were calculated using SPSS (version 22.0; IBM Corporation, Armonk, NY, USA) and software (Version 4.1.1, http://www.r-project.org) for Windows.

NPV = negative predictive value, PPV = positive predictive value.

## 4. Discussion

In this study, we established a 9-gene mutation panel based on BRAF^V600E^, NRAS (codons 12, 13, 59, 61), HRAS (codons 12, 13, 61), KRAS (codons 12, 13, 59, 61, 117, 146), TERT, TP53 (Arg273), PAX8/PPARG, CCDC6/ RET and NCOA4/ RET. The above 9-gene signatures are described as definite biomarkers with diagnostic significance in the National Comprehensive Cancer Network guidelines, which has been verified as driving genes with alternation frequency in thyroid tumors. The results of the study found that the combination of cytology and multi-gene testing greatly improved the diagnostic efficacy in distinguishing benign and malignant thyroid nodules, with diagnostic sensitivity (68.6% to 89.3%), specificity (87.5% to 95.2%), PPV (95.9% to 98.9%) and NPV (39.8% to 64.5%), respectively.

Gene mutation was detected in 324 FNA samples (324/615, 52.7%), with an overall malignancy rate of 89.7% and the incidence of lymph node metastasis of 64.7%. As expected, the mutation in BRAF gene was the most prevalent (233/324, 71.9%), which is substantially higher than that in Westerners.^[15]^ In addition, the rate of RAS gene mutation was 23.8%, with over a half of them being NRAS gene mutations, which was consistent with the existing studies.^[17–19]^

A BRAF^V600E^ -based assay has 100% specificity, although its sensitivity is limited by the restricted expression of this oncogene to PTC. Now BRAF mutation is considered to be a diagnostic marker that is useful for patients with indeterminate FNBC to help thyroid nodule management.^[20]^ Besides, BRAF^V600E^ has been considered the best candidate as molecular prognosticator of PTC, and the majority of studies reported the association of mutated BRAF with several other clinicopathological features having negative prognostic impact, such as lymph node metastases, extrathyroidal extension and advanced disease stage.^[21]^ In this study, the BRAF-mutated nodules, 99.2% being PTC, and only 1 sample was pathologically diagnosed as FA, the reason might be attributed to the low incidence of false positives. Chen et al^[22]^ found 5 false positives in total 292 samples; Kim et al^[23]^ reported 6 out of 126 samples as false positives; and Yoon Young Cho et al^[14]^ detected only 1 false positive in 293 mutated samples. Among the cases with BRAF mutations, the incidence of lymph node metastasis is 68.3%, significantly higher than other gene mutation groups. This suggests that BRAF mutations may be associated with poor prognosis.

RAS gene mutation was detected in 77 FNA samples, which was shown as the second most common mutation in this study. The reported malignancy rate of RAS-mutated nodules was 10–62%.^[24,25]^ Here, 45.8% of the RAS-mutated nodules were malignant, including 9 PTCs (4 follicular-variants, 44.4%), 1 FTC and 1 NIFTP, which is similar to most of the related reports.^[26–28]^ In addition, there were 5 NRAS-mutated nodules in the total 6 developing lymph node metastasis, suggested the relationship between NRAS mutation and lymph node metastasis. This study also found that nodules with combined BRAF and RAS mutations were all malignant PTCs.

TERT promoter mutation is accompanied by enhanced telomerase reverse transcriptase activity and increased invasion potential in tumor cells, which tremendously affect the malignancy and prognosis of thyroid cancer. In this study, TERT mutations were found in 4 nodules, of which 3 malignant nodules were 2 PTC and 1 FTC.

RET gene rearrangements were reported to occur in 6–30% PTC cases and are more prevalent after radiation exposure and/or in children.^[29,30]^ Here, we detected RET gene rearrangement only in 2 nodules, with one being PTC and the other being FA (with combined RAS gene mutation).

PAX8/PPARG rearrangements are present in classic follicular carcinoma, follicular-variant-PTC and OCC, suggesting potential implications in tumor evolution, invasion and metastasis. In this study, there were 3 cases of follicular carcinomas, 4 cases of follicular-variant-PTCs and 2 cases of OCCs. However, no PAX8/PPARG rearrangements were detected in any of these disease entities. As previously reported by Song et al^[31]^ found that the PAX8/PPARG gene mutation rate in Asians was much lower than in westerners. Consistently, PAX8/PPARG gene mutations were not reported in the multi-gene study by Yoon Young Cho et al.^[14]^

TP53 gene mutation can be detected in 50% to 80% of undifferentiated thyroid carcinoma and 7% to 25% in OCCs,^[32–34]^ demonstrating its association with the dedifferentiation of thyroid cancer. Our study did not find any TP53 mutation, which might be explained by the very limited cases of OCC and no cases of undifferentiated carcinoma.

Moreover, combined gene mutations, mainly BRAF + RAS/TERT (n = 9), were detected in 11 nodules, pathologically diagnosed as malignancy. In general cases, RAS and BRAF gene mutations are mutually exclusive, indicating the similar mutation pattern of the 2 genes with independent significance in PTC. Nevertheless, combined mutations in BRAF and RAS or other genes were previously reported in PTC cases, especially in advanced or recurrent cases.^[35–37]^ For instance, Zou et al^[35]^ reported combined BRAF and KRAS/RET gene mutations in 11 (13%) out of 88 PTC samples, the majority of which were in an advanced stage. This study also indicates that dedifferentiation is likely not driven by BRAF or RAS mutations individually but rather by the combined effort of multiple genetic alterations.^[35,38]^ Vincenzo Marotta et al^[21]^ stated that prognostic effect related to alterations of TERT promoter disappeared or strikingly decreased when mutations occurred separately, suggesting that actual prognostic value of the genetic marker had been overestimated and co-existence of BRAF mutation was mandatory for promoting tumor aggressiveness.

RAS mutation is the second most prevalent mutation type with substantial clinical significance. It can be used as a predictive marker for cancer cell subtypes and well-differentiated cancer foci yet with the potential for metastasis and dedifferentiation. Since RAS is involved in the evolutionary processes from benign FA to low-grade malignant NIFTP and even more malignant follicular carcinoma,^[39]^ RAS mutation rate is essential for clinical diagnosis, especially for the samples which cannot be determined cytologically.^[40]^ In the present study, RAS mutation comprised 17.4% of the Bethesda III/IV/V samples, making it the second most common mutation type after BRAF (Table 3). It also explains why the 9-gene mutation panel used in testing yielded higher diagnostic sensitivity but lower specificity than cytology alone. When compared with the 7-gene mutation panels reported by Nikiforov et al^[11]^ and Yoon Young Cho et al,^[14]^ the 9-gene mutation panel in this study resulted in a low rate of benign tumors (13.2%) while a relatively high rate of mutation (17.4%), which would be a disadvantage for applying 9-gene testing in cytologically indeterminate samples.

Previous research revealed that the malignancy of RAS mutation ranges between 10% and 62%,^[24,25]^ taking into account differences in histopathological criteria across institutes and research groups. Notably, diagnosis for low-grade malignancies such as NIFTP differs in terms of capsular invasion, nuclear features and proportion of true papillae.^[41]^ Our study reported that 45.8% of RAS-mutated thyroid nodules were malignant, including 9 PTCs, 1 FTC and 1 NIFTP. The result is similar to the multi-center study of Steward, D.L. et al,^[24]^ in which 57% of RAS-mutated nodules were predicted as cancer or NIFTP. Despite the wide range of malignancy rate of RAS-mutated thyroid nodules, gene testing with a combined use of ultrasound and cytological grading still provides an essential reference for clinical diagnosis and decision-making. Some believe that the risk of malignant transformation in RAS-mutated, histopathological-diagnosed benign nodules remains increased, and surgical resection appears to be the optimal treatment option. Evidence suggests that a select group of patients with RAS-positive well-differentiated thyroid cancer may be at risk for RAS-mediated tumor dedifferentiation, distant metastases, and shortened survival.^[42,43]^ Fukahori et al^[44]^ reported that RAS was significantly associated with distant metastasis and death in their series of patients with FTC. Marotta V et al^[45]^ found that RAS mutations are not relevant in the diagnostic definition of thyroid nodules, but may provide a surgical indication due to the relationship of high growth rate in benign nodules. Puzziello A et al^[46]^ also stated that although mutated Ras does not enable a diagnosis of malignancy, its presence increases the risk of malignancy of indeterminate nodules, thus suggesting a more strict surveillance or surgery, according to clinical evaluation. These studies further indicate that RAS-mutated nodules should be clinically valued, whether benign or malignant in morphology.

In this present study, we focused on surgically resected samples, as histopathology of these cases provides the gold standard to define the nature of the tumors. Since all surgical decisions were made after ultrasound examination, cytological grading, and multi-gene testing, the malignancy rate in this study was up to 83%, much higher than in other studies (malignancy rate 15–35%).^[8,9]^ When combined with cytology, the specificity, PPV and NPV were up to 95.2%, 98.9% and 64.5%, respectively. Compared to Sylvie Beaudenon-Huibregtse study, NPV was lower, but the specificity and PPV were higher.^[7]^ These results suggest that most thyroid nodules can be well-diagnosed before surgery.

In recent years, molecular testing for oncogenic gene mutations plays agrowing role in the optimal management of thyroid nodules, common panel of mutations observed in various study. Yuri E. Nikiforov. et al established a 7-gene mutation panel and indicated that molecular analysis for a panel of mutations has significant diagnostic value for all categories of indeterminate cytology and can be helpful for more effective clinical management of these patients.^[47]^ Erik K. Alexander study suggested that consideration of a more conservative clinical approach for patients who have nodules with indeterminate cytologic features on fine-needle aspiration and a benign result on gene-expression classifier testing.^[48]^ There is compelling evidence indicating that single gene (BRAF) testing exhibits high specificity and PPV in the diagnosis of PTC, while demonstrating low sensitivity and NPV. The objective of preoperative molecular diagnostics for thyroid nodules is to enhance the NPV by expanding the gene panel, thereby minimizing unnecessary surgeries. Admittedly, due to the selectivity of the cases, the 9-gene mutation panel cannot serve as an independent and random tool for assessing thyroid nodules. In addition, our study detected gene mutations in 324/615 nodules, but only 209 (209/324, 34%) nodules were surgically removed in our hospital. The tumor types diagnosed were PTC, FTC, OCC, NIFTP and HTT, among which a very small number of cases were NIFTP and HTT. No other tumor types were found. All the above limitations inevitably impair the completeness and accuracy of our data. Therefore, a more comprehensive range of cases are warranted in our future study.

In conclusion, our study established a 9-gene mutation panel and combined it with cytology to diagnose thyroid nodules, resulting in improved specificity and PPV. Our strategy avoids unnecessary surgeries or second surgery and can be used as an essential reference for clinical decision-making and prognosis assessment. In the future, the diagnostic performance of the 9-gene mutation panel will be further evaluated and improved.

## 5. Conclusion

Our study established a 9-gene mutation panel and combined it with cytology to diagnose thyroid nodules, resulting in improved specificity and NPV. Our strategy avoids unnecessary surgeries or second surgery and can be used as an essential reference for clinical decision-making and prognosis assessment. In the future, the diagnostic performance of the 9-gene mutation panel will be further evaluated and improved.

## Author contributions

**Conceptualization:** Murui Zhang.

**Data curation:** Murui Zhang, Xiaotong Hu.

**Formal analysis:** Murui Zhang, Junchang Jiang, Hui Li, Weiqiang Fei, Tingting Zhong.

**Project administration:** Murui Zhang.

**Software:** Murui Zhang, Hui Li.

**Supervision:** Zhinong Jiang.

**Writing – original draft:** Murui Zhang.

**Writing – review & editing:** Murui Zhang, Xiaotong Hu, Lunming Liu, Yihong Wang.
